# Applications of stable, nonradioactive isotope tracers in *in vivo* human metabolic research

**DOI:** 10.1038/emm.2015.97

**Published:** 2016-01-15

**Authors:** Il-Young Kim, Sang-Hoon Suh, In-Kyu Lee, Robert R Wolfe

**Affiliations:** 1 Department of Geriatrics, the Center for Translational Research on Aging and Longevity, Donald W. Reynolds Institute on Aging, College of Medicine, The University of Arkansas for Medical Sciences, Little Rock, AR, USA; 2 Deparatment of Physical Education, College of Sciences in Education, Yonsei University, Seoul, Republic of Korea; 3 Section of Endocrinology, Department of Internal Medicine, School of Medicine, Kyungpook National University, Daegu, Republic of Korea

## Abstract

The human body is in a constant state of turnover, that is, being synthesized, broken down and/or converted to different compounds. The dynamic nature of *in vivo* kinetics of human metabolism at rest and in stressed conditions such as exercise and pathophysiological conditions such as diabetes and cancer can be quantitatively assessed with stable, nonradioactive isotope tracers in conjunction with gas or liquid chromatography mass spectrometry and modeling. Although measurements of metabolite concentrations have been useful as general indicators of one's health status, critical information on *in vivo* kinetics of metabolites such as rates of production, appearance or disappearance of metabolites are not provided. Over the past decades, stable, nonradioactive isotope tracers have been used to provide information on dynamics of specific metabolites. Stable isotope tracers can be used in conjunction with molecular and cellular biology tools, thereby providing an in-depth dynamic assessment of metabolic changes, as well as simultaneous investigation of the molecular basis for the observed kinetic responses. In this review, we will introduce basic principles of stable isotope methodology for tracing *in vivo* kinetics of human or animal metabolism with examples of quantifying certain aspects of *in vivo* kinetics of carbohydrate, lipid and protein metabolism.

## Introduction

**Figure 1:**
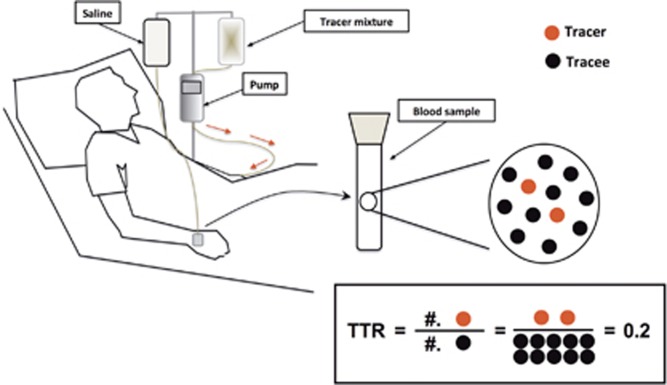
Schematic diagram of human stable isotope infusion study. It is typical that one or more stable isotope tracers are introduced intravenously into the systemic circulation via a catheter placed on one arm. Blood (or other accessible tissues such as muscle and adipose tissues) samples are collected from the other arm (or hand) before and after the tracer infusion. Isotopic enrichment (tracer to tracee ratio, or *TTR*) of a compound of interest is subsequently determined by means of GC/MS.

The human body is in a dynamic homeostasis. In other words, all aspects of the body are in a state of turnover, that is, being synthesized, broken down and/or converted to different compounds. For example, the muscle protein pool size in the body is relatively constant in healthy adults because the continual breakdown of muscle protein is matched by a corresponding synthesis of new muscle protein. A change in pool (for example, protein) size reflects either net synthesis, that is, synthesis exceeding breakdown (for example, muscle hypertrophy) or net breakdown, that is, breakdown exceeding synthesis (for example, cancer cachexia and sarcopenia). This dynamic nature of various aspects of human metabolism can be best explored *in vivo* by the use of stable, nonradioactive tracers with the help of gas or liquid chromatography mass spectrometry (GC/MS or LC/MS). Figure 1 shows a schematic diagram of a human subject participating in a stable isotope tracer infusion. In general, one or more stable isotope tracers are introduced intravenously into the systemic circulation. Most commonly, blood (or other accessible tissues such as muscle and adipose tissues) samples are collected before and during the tracer infusion. Isotopic enrichment is subsequently determined by means of GC/MS or LC/MS. And finally, the *in vivo* kinetics of the substance being traced (‘tracee') is calculated on the basis of the measured ratio of the tracer to tracee in the samples (for example, fractional synthesis rate, *FSR*) or in conjunction with the tracer infusion rate (*F*) (for example, rate of appearance, *R*_*a*_).^1^ In this review, we will briefly discuss the following topics: the meaning of metabolic isotope tracer, determination of isotopic enrichment, utilization of GC/MS, performance of a tracer infusion study, mathematic models for estimation of *in vivo* kinetics and biological applications of the tracer techniques. We also provide exemplary calculations of carbohydrate, lipid and protein kinetics as Supplementary Data (S1–6). Our goal is to provide a basic overview for investigators not familiar with the methodology. More comprehensive and detailed reference sources are available.^1, 2^ In addition, there is a National Institute of Health/Mouse Metabolic Phenotyping Centers (NIH/MMPC)-sponsored annual Isotope Tracers in Metabolic Research course (https://www.mmpc.org/shared/tracers.aspx).

## Metabolic isotope tracers

A metabolic isotope tracer is a molecule that is chemically and functionally identical to the naturally occurring molecule of interest (that is, tracee), and following the fate of the tracer provides information on the metabolism of the tracee.^1^ A metabolic tracer can be a molecule labeled with a stable or radioactive isotope(s). However, potential adverse health effects of radioactivity in human subjects have limited the use of radioactive tracers. In addition, the versatility of stable isotope methodology has greatly expanded the variety of studies possible. Therefore, this review will focus on the applications of stable isotope tracers to explore *in vivo* kinetics of human metabolism.

### Stable isotopes

**Figure 2:**
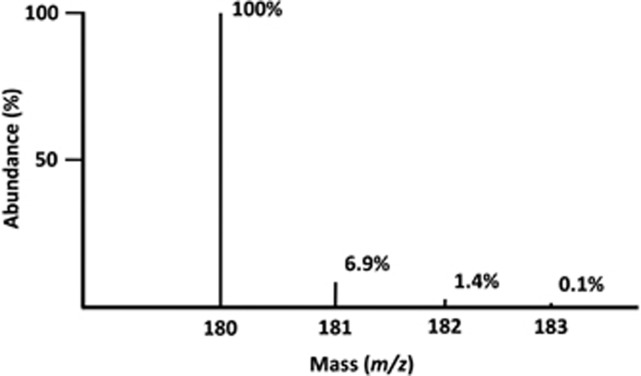
Distribution pattern of abundance of naturally occurring glucose with increasing mass as a result of presence of heavier isotopes of carbon, hydrogen and oxygen (sources from Scientific Instrument Services. www.sisweb.com).

Isotopes are elements that are chemically identical but different in mass because of the differences in the number of neutrons in their nuclei, and are either radioactive or stable, nonradioactive isotopes. As opposed to radioactive isotopes, stable isotopes are naturally occurring and stable over time without undergoing spontaneous decay with emission of radiation. In human metabolic research, the most commonly used stable isotopes include hydrogen, carbon and nitrogen isotopes that can be incorporated into molecules to be used as metabolic tracers. These elements have one or more isotopes with specific natural distribution patterns of abundance. For instance, carbon has two stable isotopes, that is, carbon 12 (^12^C) and carbon 13 (^13^C), with natural occurrence of approximately 98.9% and 1.1%, respectively. Owing to the existence of isotopes, there exists a unique distribution pattern of naturally occurring molecules with varying masses. Figure 2 shows the relative abundance of naturally occurring glucose with increasing masses. Because the glucose molecule has six carbons, and each carbon has approximately 1.1% of chance to be ^13^C, 6.6% of naturally occurring glucose will be glucose with having at least one ^13^C. When the abundance of the most abundant form of glucose molecule (typically, the lowest mass, *M+0*; molecular weight 180) is normalized to 100%, relative abundance of glucose with molecular masses of 181 (*M+1*), 182 (*M+2*) and 183 (*M+3*) are 6.9%, 1.4% and 0.1%, respectively (Figure 2).

### Metabolic tracers

A stable isotope tracer is a molecule with one or more isotopes with a different mass than the most abundantly occurring mass incorporated somewhere in the molecule. In the case of the most commonly used tracers (that is, C, H and N), the stable isotope tracers are heavier than the most commonly occurring mass. The fact that the tracer is heavier than the tracee allows for determination of the ratio of tracer to tracee (that is, enrichment) by measuring the mass of the molecule of interest. Information on the ratio is generally obtained by using GC/MS, but LC/MS can also be used. The general principles are similar, regardless of the means of measurement; so in this review, we will focus on the more commonly used approach of GC/MS. Regardless of the means of analysis, if a tracer such as 1-^13^C-glucose is infused intravenously into the systemic circulation for the determination of *R*_*a*_ glucose in the fasted state, the 1-^13^C-glucose is heavier than the most abundant form of naturally occurring glucose (containing only ^12^C, designated as *M+0*) by 1 mass unit (that is, *M+1*). Measurement of the mass differences between tracer and tracee (unlabeled and 1-^13^C-glucose in this example) enables the calculation of *in vivo* kinetics of the tracee. Calculations must account for the existence of naturally occurring heavier isotopes in many elements as discussed above. Thus, some portion of glucose is already *M+1* (for simplicity, we ignored heavier isotopes of hydrogen and oxygen here). This naturally occurring heavier glucose (*M+1*) cannot be differentiated from the *M+1* glucose tracer that has been infused (for example, 1-^13^C-glucsoe, or glucose with one heavier carbon isotope in any place). However, this can be typically resolved by subtracting background enrichment from the enrichment of the sample collected after infusion of tracer. There are additional factors that must be accounted for when performing kinetic calculations in stable isotope tracer studies such as skew correction and overlapping spectra,^3^ but these issues are beyond the scope of this paper. Using metabolic tracers, we can trace many aspects of *in vivo* metabolism. To understand how to determine *in vivo* kinetics of metabolism, it is first necessary to understand how isotopic enrichment is determined, and the enrichment information is used to calculate *in vivo* kinetics.

## Determination of isotopic enrichment

**Figure 3:**
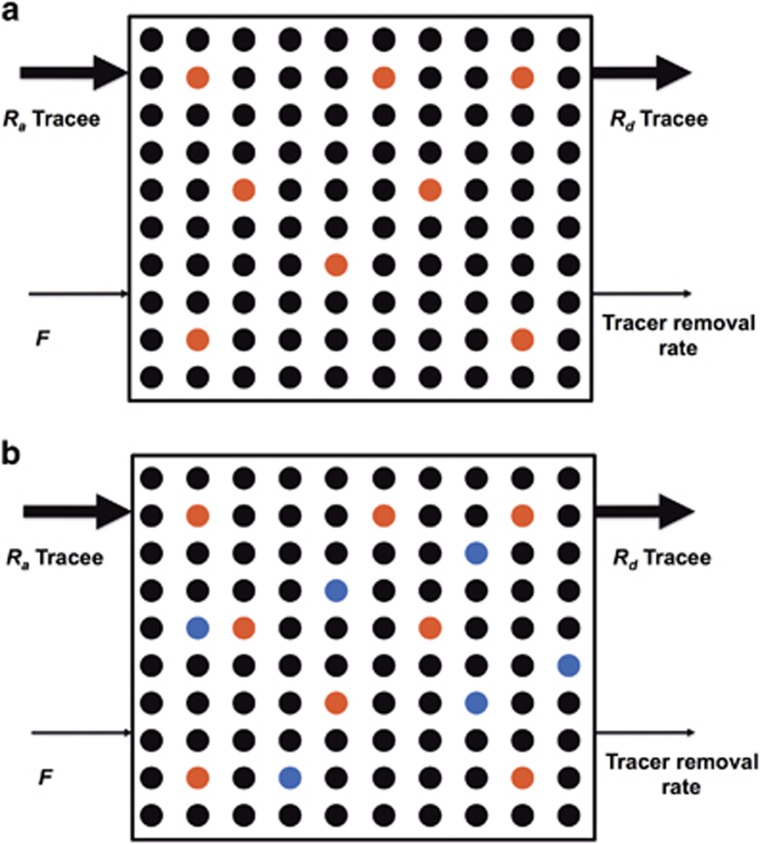
Conceptual schematic diagrams of isotopic enrichment. The systemic circulation is depicted as rectangular boxes. Assume that one kind of compound (e.g., glucose) exists in circulation, and singly labeled glucose tracer (e.g., 1-^13^C-glucsoe, *M+1*) is administered. (**a**) Enrichment (e.g., tracer to tracee ratio, *TTR*) is simply the ratio of concentrations of tracer (red circle) to tracee (black circle), which is the ratio of 8 to 92 (*TTR*=0.087). (**b**) Some proportion of tracee is actually heavier than the most abundant form (typically, *M+0*). For example, 6.9% of naturally occurring glucose has mass of 181 (*M+1*, blue circle) when *M+0* glucose is normalized to 100%. Because MS does not distinguish *M+1* of tracee from *M+1* of tracer, correction needs to be made by subtracting background enrichment from sample enrichment.

Figure 3 depicts the relationship between tracer and tracee in a steady state during a continuous tracer infusion. In a metabolic study, one or more of isotope tracers are infused into the body intravenously and then samples are frequently collected from which relative concentrations of tracer to tracee (that is, isotope enrichment) are quantified. The ratio of tracer to tracee is used for the calculation of tracee kinetics. Although there are several ways to express isotopic enrichment,^1^ tracer to tracee ratio (*TTR* or *t/T*) is the most widely used. The expression of isotopic enrichment as *atom percent excess* or *mole percent excess* (*MPE*) is also common. These later expressions of enrichment are derivatives of *TTR*, as both *atom percent excess* and *MPE* (%) are expressed as *TTR*/(1+*TTR*) × 100. *Atom percent excess* and *MPE* are typically used when information on total *R*_*a*_ (*R*_*a*_ tracee+*R*_*a*_ tracer) is needed as in the case for the calculation of glucose kinetics,^3, 4, 5^ protein synthesis^6^ and substrate oxidation.^7^ Exemplary calculations of isotope enrichment are shown in Supplementary Data (S1). This example illustrates determination of isotope enrichment of 6,6-^2^H_2_-glucose tracer (a glucose tracer in which two hydrogens attached to six carbons are deuterium) from blood samples collected before and after the tracer infusion, and samples are analyzed using GC/MS. In the following section, we will briefly discuss how GC/MS is used to determine enrichment.

## Gas chromatography mass spectrometry

**Figure 4:**
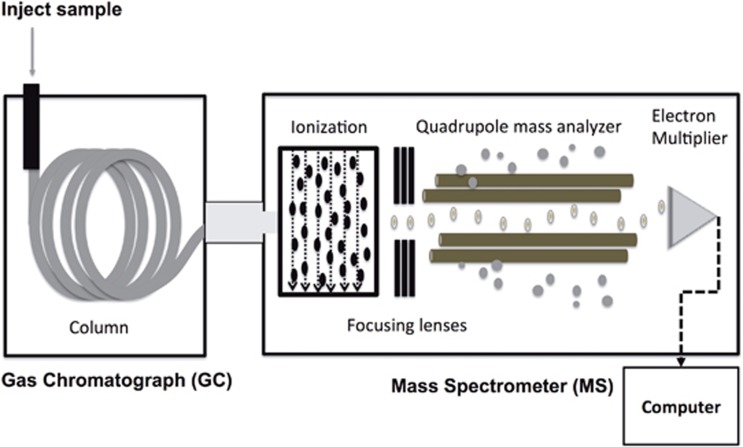
Schematic diagram of a gas chromatography quadrupole mass spectrometer (GC/MS).

Figure 4 depicts a schematic representation of a GC/MS. Here, we briefly introduce how to utilize GC/MS for the determination of isotopic enrichment.^1^ For more comprehensive information, refer to other sources.^8, 9, 10^

### Gas chromatography (GC)

The primary function of GC is to separate the compound of interest from others in a sample mixture and to introduce one compound at a time into the MS. Before injection into the GC, it is typical to process a sample mixture for better GC/MS results, including chemical derivatization of the compound.^11^ The derivatization is necessary to make the sample volatile with modest heating for GC analysis. The processed sample mixture is then injected into GC where individual compounds in the mixture migrate at different rates through the GC column via a carrier gas (typically, helium) depending on their chemical characteristics.^9^

### Mass spectrometry (MS)

**Figure 5:**
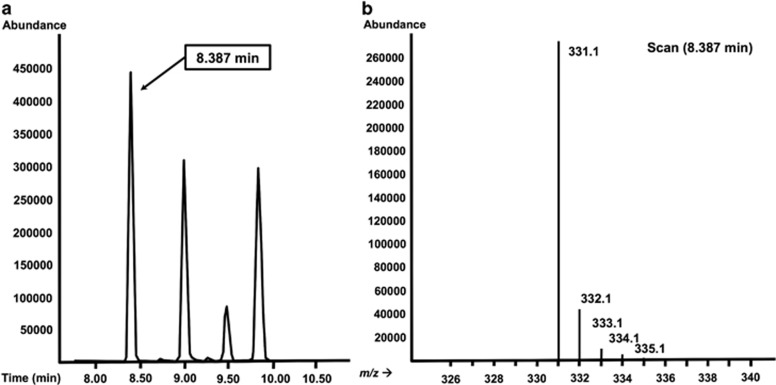
(**a**) Total ion chromatogram (TIC), each peak generally representing different compounds, eluting from GC at different time and (**b**) mass spectrum, showing abundance of ions with varying masses (heavier toward right) due to fragmentations of the compound, each peak from TIC is the sum of abundance from all the ions in its mass spectrum.

The primary function of MS is to quantify the abundance of a specific mass of ion that is fragmented from either unlabeled or labeled compounds of interest in a process of ionization in ion source of the MS via either electron impact ionization (generating fragmentations to a great extent) or chemical impact ionization.^1^ After passing through the ionization source, fragmented ions with specific mass/charge (*m/z*) values can travel through the mass analyzer (or mass filter) for a given set time and hit the electron multiplier (or photomultiplier).^1^ For biological samples, the charge is generally one, so samples are separated entirely on the basis of mass. The photomultiplier generates a signal, the intensity of which is directly proportional to the abundance of the ions.^9^ Figure 5 shows the total ion chromatogram and mass spectrum of ions of the pentaacetate derivative of glucose after chemical impact ionization. For determination of isotopic enrichment, a good ion fragment for monitoring must be identified. Then, the selected ion fragment can be monitored using selected ion monitoring.^9^ As its name implies, selected ion monitoring monitors only a few selected number of masses from fragmented ions rather than monitoring the ions for all *m/z*, which increases specificity and thus improves the detection limit.^9^ In stable isotope research, infusion of an isotope tracer with one or more of labels (for example, 1-^13^C-glucsoe, *M+1*) will increase the abundance of ions with a mass increase (*M+1*). In this case, *TTR* will be the background-subtracted ratio of *M+i* to *M+0* (*M+i*, molecule with *i* mass unit above *M+0*). For more detailed explanation, refer to additional sources.^1, 12^

### Isotope ratio MS

The isotope ratio MS is commonly used to analyze enrichment of gases such as N_2_, O_2_ and CO_2_.^1^ In metabolic studies, the isotope ratio MS is typically used for the determination of ^13^CO_2_ enrichment in the breath sample collected after administration of ^13^C-labeled substrate such as 1-^13^C-glucose, U-^13^C_6_-glucose, 1-^13^C-palmitate and U-^13^C_16_-palmitate (the U- refers to the fact that the molecule is uniformly labeled, meaning that all carbons are enriched with carbon 13) for estimating the rate of substrate oxidation in conjunction with indirect calorimetry. For example, breath CO_2_ enrichment can be determined (ratio of ^13^CO_2_ to ^12^CO_2_) after infusion of a ^13^C-labeled tracer to determine the rates of oxidation of specific components of carbohydrate (for example, glucose)^13, 14^ and lipids (for example, oleate and palmitate).^5, 15^ The rate of oxidation of individual amino acids (AA) that can be oxidized in tissues such as muscle (that is, leucine) can also be determined with ^13^C-labeled tracers.^16, 17^ We will provide an example of the calculation of substrate oxidation later in this review (Supplementary Data (S6)). Before this, we will touch briefly on the mathematical models used for the estimation of kinetics of various tracees.

## Mathematical models

In a general sense, the mathematical models used to calculate tracee kinetics can be collectively divided into two main categories: (i) tracer dilution model and (ii) tracer incorporation model.

### Tracer dilution model

The dilution model can be useful for the determination of tracee kinetics both in a steady state and in a nonsteady state. In a physiological steady state, for example, glucose concentrations are constant over time, which means that the rate that glucose appears (*R*_*a*_ glucose) into blood from all sources is equal to the rate that glucose disappears (*R*_*d*_ glucose) from the blood. With a primed continuous infusion, the magnitude of dilution of this isotope tracer by tracee in the plasma compartment at an isotopic steady state reflects *R*_*a*_ tracee of interest in plasma (in this example, glucose). This calculation does not account for the total production (if any) of tracee in compartments where the tracee is produced without appearing into plasma.^1, 2^ The dilution model has been widely used for the calculation of tracee, including glucose,^3, 4, 5, 13, 14, 18^ fatty acid,^5, 15, 18^ glycerol^7, 19^ and lactate^20, 21^ in steady states and nonsteady states. Contrary to steady-state conditions, it is more complex to calculate the kinetics in nonsteady-state conditions such as during a meal feeding or exercise. The most important calculation for nonsteady-state kinetics was carried out by Steele in 1959 to investigate glucose metabolism in a situation where insulin was injected.^22^ The so-called ‘Steele' equation has been extensively used for nonsteady-state calculations of *in vivo* kinetics of glucose,^3, 4, 13, 14^ free fatty acids (FFA),^5, 15, 23^ glycerol^23, 24^ and lactate,^20, 25^ since that pioneering study. The Steele equation is derived for the use with compounds that are either distributed in a single pool, or can be described as if the sampled compartment represents a fraction of a single pool. Because of the complexity of calculating kinetics in the nonsteady state, we will focus mainly on the use of the tracer dilution model for steady-state conditions.

#### Rationale for calculation of rate of appearance of tracee in steady-state conditions

**Figure 6:**
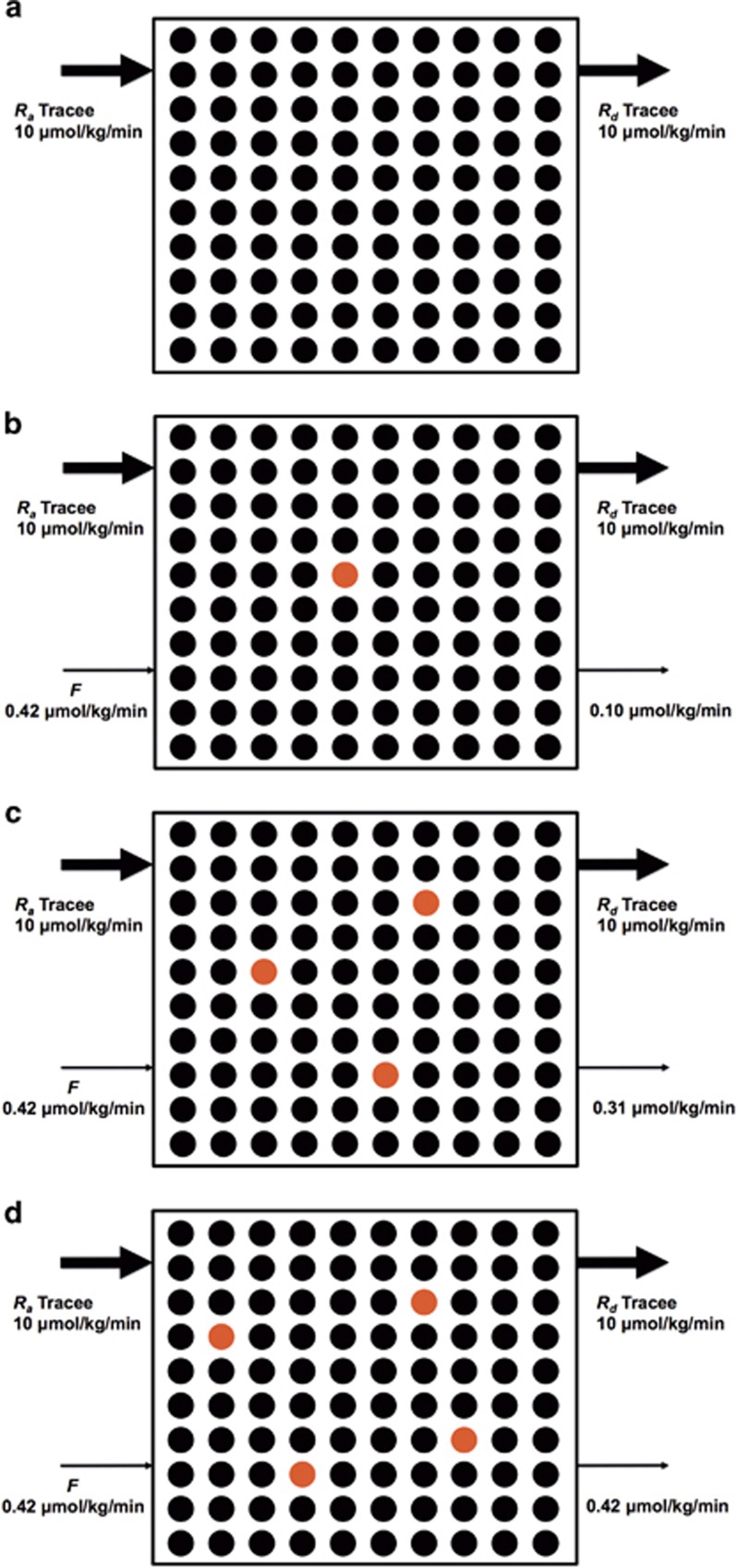
Schematic rationale for the determination of *R*_*a*_ tracee using a stable isotope tracer method in a physiological steady state. Before (**a**) and throughout a tracer infusion (**b**, **c** and **d**), *R*_*a*_ tracee will be equal to *R*_*d*_ tracee, both of which are being constant in a steady state (**d**). To estimate *R*_*a*_ tracee, a tracer is primed continuously infused intravenously. At the beginning of the infusion, rate of removal of tracer from the compartment (**b** and **c**) is lower than tracer infusion rate (*F)*. However, it will be eventually equal over time to *F* (**d**). Red and black circles represent tracee and tracer, respectively. For simplicity, naturally occurring heavier glucose molecules were ignored.

In a steady state, the blood concentration of the tracee of interest is constant. This is because the rate at which the tracee appears in the blood is equal to the rate at which it disappears from the blood. Figure 6 is a schematic representation of the relationship between *R*_*a*_ tracee, *F* and *TTR* in a physiological steady state. Figure 6a depicts a condition before tracer infusion with the assumption that *R*_*a*_ glucose is 10 μmol kg^−1^ min^−1^. In an actual experiment, *R*_*a*_ glucose would be the unknown and it would be calculated using tracer methodology. As *R*_*a*_ is equal to *R*_*d*_ in a steady state, we know in this example that *R*_*d*_ glucose must also be 10 μmol kg^−1^ min^−1^. To determine *R*_*a*_ glucose, a glucose tracer is started at a rate (for example) of 0.42 μmol kg^−1^ min^−1^ (Figure 6b). Several general assumptions in this model must be satisfied:^1^ (i) the infused tracer does not affect the endogenous metabolism of tracee of interest, (ii) the infused tracer does not reappear in the sampled compartment (for example, blood) to which the tracer initially infused and then removed from the compartment, and (iii) the body does not distinguish between tracer and tracee, and therefore removal of the tracer and tracee depends on their relative concentrations in the compartment, and others. In Figure 6b, although *R*_*a*_ glucose is equal to *R*_*d*_ glucose, *F* is higher than the rate of tracer removal from the sampled compartment at the beginning of the tracer infusion. However, the rate of tracer removal will eventually become equal to *F* (Figure 6d). Over time, *TTR* will increase as seen in Figure 6 (order of time sequence: 6a→6b→6c), and then will plateau when *F* is equal to the rate of tracer removal rate (Figure 6d), at which it is called enrichment at isotopic equilibrium, or plateau enrichment (*E*_*p*_). Once the *F* and *E*_*p*_ are known, *R*_*a*_ tracee can be calculated by dividing F by *E*_*p*_. For a given *F*, *R*_*a*_ tracee will be inversely related to *E*_*p*_, such that *R*_*a*_ tracee will be higher if the measured value for *E*_*p*_ is lower. Although in this schematic, we describe the relations with a continuous tracer infusion, the basic idea will be the same with injection of a priming dose. In the case of the primed continuous infusion of tracer, the time to achieve *E*_*p*_ is reduced. An appropriate priming dose can be determined from previous experiments reported in the literature, or by performing a preliminary study before start of a new stable isotope tracer study. The approach to calculating an appropriate priming dose is described elsewhere.^1^

#### Derivation of steady-state kinetic equations

Assuming that the infusion of a tracer does not affect endogenous metabolism of the tracee of interest, the relation between *R*_*a*_ tracee, *F* and *TTR* at a steady state can be mathematically derived:^1^


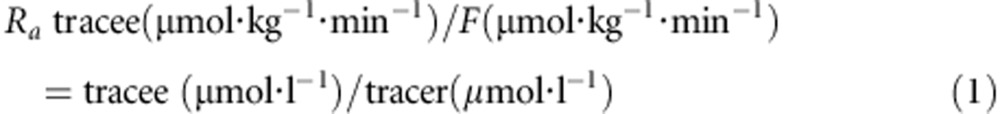


After rearranging the equation (1) for *R*_*a*_ tracee,


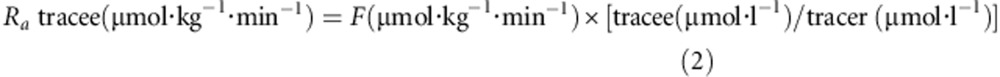


By canceling the units of denominator and numerator on the right part of the equation [tracee (μmol l^−1^)/tracer (μmol l^−1^)] equation (2), and because reciprocal of tracee/tracer is *TTR*, the equation (2) can be written as:


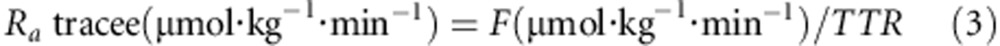


Because *TTR* at isotopic equilibrium is called plateau isotopic enrichment (*E*_*p*_), the equation (3) can be written as:


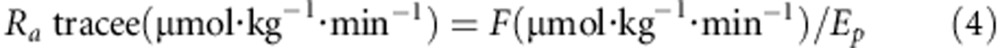


### Tracer incorporation model

The incorporation model is based on the rate of incorporation of precursor tracer into product over a given time. This yields a *FSR*, which is commonly expressed as %/h. This value (after converting from % to fractional value) must be multiplied by the total pool size to derive an absolute synthesis rate. As with any model of kinetics, there are several assumptions with the incorporation model, including that the pool size of the product and rate of incorporation of precursor for the early time after the start of the tracer infusion is constant.^1, 2^ Many investigators have used *precursor-product models* to estimate *FSR*.^2, 26^ For example, when determining the rate of protein synthesis, precursors are AA and proteins are products. Using this incorporation model, the kinetics of various compounds can be determined including *FSR* of individual proteins^27^ and subgroups of proteins (mitochondrial, sarcoplasmic and mixed muscle protein),^6, 28^ substrate oxidation rate,^5^ DNA synthesis rate (thus cell division)^29, 30^ and nitric oxide synthesis rate from the precursor arginine.^31, 32^

## Performing isotope tracer infusion study

### Catheterization

Before starting a tracer infusion study, catheters are generally placed in peripheral veins in the arms: one for infusion of tracers and the other in the contralateral arm for blood sampling. However, when a sample needs to come from an artery such as in an arteriovenous balance study, a catheter needs to be placed directly in an artery.^33^ Arterial catheterization has certain inherent risks, particularly in vulnerable populations.^34^ Alternatively, the ‘heated-hand' technique in which a hand vein is typically warmed to ~65 °C for ~10 min can be used to obtain an ‘arterialized' blood sample, which enables the measurement of values that approximate those in true arterial blood.^35, 36^

### Tracer administration method

Upon appropriate catheterization, one or more of tracers can be introduced into the circulation by either oral ingestion or intravenous infusion or injection. Intravenous administration is most common. The most commonly used method is the ‘primed continuous (constant) infusion' method. In this method, an appropriate priming dose of tracer is intravenously injected as a bolus to hasten achieving a plateau in enrichment, followed by a continuous infusion at a rate that is generally chosen to produce *E*_*p*_ of less than 0.10. A greater abundance of tracer could potentially alter the endogenous metabolism of tracee. It is optimal to achieve enrichment as low as possible within the detection limits of the GC/MS, thereby reducing the possibility of altering the endogenous metabolism of the tracee. A lower infusion rate also has the benefit of requiring less tracer, thereby reducing the cost. Without priming, *E*_*p*_ will still be achieved eventually, but it will take a much longer time. Lengthening the infusion time may present logistical problems in human research, and also impact the physiological state. After finishing an infusion study, isotopic enrichment in the samples will be determined and used for the calculation of kinetics of the tracee of interest. In the following section, we will briefly discuss some of the biological aspects that we could trace with use of stable isotope tracer.

## Biological applications

Here, we briefly discuss the biological applications of stable isotope tracer methodology with respect to *in vivo* dynamics (kinetics) of carbohydrate, lipid and protein metabolism. These examples are chosen primarily for the purpose of illustrating the type of questions that can be addressed with tracer methodology.

### Glucose kinetics

Under normal conditions, the blood glucose concentration in the fasted state in healthy individuals is fairly constant over time, which is accomplished through a close matching between rate of appearance (*R*_*a*_) in and rate of disappearance (*R*_*d*_) of glucose from the circulation. In clinical conditions such as obesity and type 2 diabetes mellitus, however, alterations in glucose kinetics are apparent.^37^ Briefly, *R*_*a*_ glucose is derived mainly from hepatic glucose production (via glycogenolysis and gluconeogenesis) and to a minor extent from renal gluconeogenesis. Fates of *R*_*d*_ glucose includes glycolysis, mitochondrial oxidation, glycogen synthesis and other metabolic pathways. These dynamics of *in vivo* glucose metabolism can be best quantified with the use of stable isotope tracer methodology. Here, we will discuss some aspects of *in vivo* kinetics of glucose metabolism. To determine glucose kinetics in a steady state, a glucose tracer (for example, 1-^13^C-glucsoe as in Example 2) is continuously infused intravenously following a bolus injection of the same tracer (priming dose). Plasma samples are collected before the tracer infusion and a couple of times after achieving isotopic equilibrium for determination of the steady-state kinetics of the tracee. Exemplary calculations of *R*_*a*_ and *R*_*d*_ glucose in a steady state are shown in Supplementary Data (S2). Owing to the existence of substrate cycles at several steps of glycolysis such as the glucose cycle during which the loss of isotope label occurs without net conversion of precursor to product, an appropriate selection of glucose tracer is crucial for the accurate quantification of glucose kinetics.^38^

### Lipid kinetics

Lipids comprise more than 80% of the body's stored fuel energy as triacylglycerols (TAG), mainly in adipose tissue. Body fat turns over continuously, and the fat mass is the direct result of the balance between synthesis and removal (that is, turnover) of TAG, and alterations in TAG turnover can lead to metabolic complications such as obesity and dyslipidemia. Briefly, TAG is broken down to three FFA and one glycerol through a series of enzyme reactions including hormone-sensitive lipase.^39^ The process is called lipolysis. Fatty acids released from TAG can be either released into systemic circulation or used for resynthesis of TAG (adipocyte re-esterification or ‘intracellular' recycling) with incorporation of glycerol phosphate generated from glycolysis in the adipocyte. Fatty acids released into the circulation are cleared by tissues where they can be oxidized into CO_2_ and H_2_O for ATP resynthesis or re-esterified into TAG (‘extracellular recycling'). Contrary to the fates of FFA, glycerol released from TAG cannot directly participate in TAG re-esterification but instead is released into the systemic circulation because of the lack of glycerol kinase in tissues other than liver, which converts glycerol to glycerol phosphate, a required step for re-esterification. Thus, *R*_*a*_ glycerol is a direct reflection of the rate of lipolysis at the whole-body level, which can therefore be quantified by tracing *R*_*a*_ glycerol into the circulation using a glycerol tracer.^1^ Further, with a simultaneous infusion of a palmitate tracer along with an infusion of a glycerol tracer, intracellular, extracellular and total (intra-+extra-cellular) recycling rates can be calculated.^40^ Exemplary calculations of lipolysis, fatty acid turnovers (*R*_*a*_ and *R*_*d*_), and substrate cycling are shown in Supplementary Data (S3). Briefly, 1-^13^C-palmitate is constantly infused without a priming dose because of the rapidly mixing plasma pool and its rapid turnover rate^41^ while primed continuous infusion of [1,1,2,3,3-^2^H_2_]-glycerol is simultaneously performed. *R*_*a*_ palmitate and *R*_*a*_ glycerol (or rate of lipolysis) are determined as the infusion rate of the respective tracer divided by plateau enrichment (*E*_*p*_) in the plasma. Then *R*_*a*_ FFA is estimated as *R*_*a*_ palmitate divided by the fractional contribution of palmitate to total FFA, which is reported to be approximately 0.65 (or 65%) at rest.^42^ Now, the intracellular recycling or re-esterification rate is determined as three times *R*_*a*_ glycerol (lipolysis) minus *R*_*a*_ palmitate, because the stoichiometry between FFA and glycerol is 3:1 per lipolysis. Extracellular recycling rate is determined as *R*_*a*_ FFA minus FFA oxidation rate (discussed later in the section Substrate oxidation and Supplementary Data (S6)). Finally, the total recycling rate is determined as the sum rates of intracellular and extracellular recycling where total recycling rate =3 × *R*_*a*_ glycerol−FFA oxidation rate.

### Protein kinetics

The amount of protein in the body (the protein pool size) is relatively constant because the rates of protein synthesis and breakdown are closely matched over time in normal healthy adults. However, the protein pool size can change as a result of normal or pathological processes, for example, resistance exercise with sound nutrition (hypertrophy), age-associated loss of muscle mass (that is, sarcopenia) or diseases (for example, cancer cachexia).^43^ Simultaneous quantifications of protein synthesis and breakdown at individual protein, tissue and whole-body levels are therefore of primary importance for understanding a complete picture of protein metabolism. For example, there is a progressive loss of muscle mass (protein) in certain clinical conditions such as burn injury and cancer cachexia as a result of protein breakdown exceeding synthesis.^43^ As opposed to our initial intuition, protein synthesis is typically elevated in these clinical conditions. This increase in protein synthesis is actually secondary to an increase in protein breakdown because AA derived from protein breakdown provides building blocks for ~80% of newly synthesized proteins.^1^ First, we will discuss a representative method among others for exploring *in vivo* whole-body kinetics of protein turnover. Here, we will discuss an essential AA (EAA) tracer method.^6^ The general approach is fundamentally based on the determination of the *R*_*a*_ into and the *R*_*d*_ from plasma of an EAA such as phenylalanine. For example, *R*_*a*_ of any EAA reflects the rate of protein breakdown in the fasted state because protein breakdown is the sole source of EAAs released into the plasma, because EAAs are not produced in the body.^1^ Therefore, in post-absorptive states, the rate of protein breakdown can be estimated by dividing *R*_*a*_ of any EAA by its fractional contribution to protein.^44^ Fates of *R*_*d*_ EAA include protein synthesis and oxidation (or hydroxylation of phenylalanine to tyrosine in the case of phenylalanine). With an additional determination of EAA oxidation in the post-absorptive state where *R*_*a*_ is equal to *R*_*d*_, the rate of protein synthesis can be quantified as *R*_*d*_ minus the EAA oxidation (or hydroxylation) rate. Exemplary calculations of whole-body protein kinetics using phenylalanine and tyrosine tracers are shown in Supplementary Data (S4).^6, 45^ In this case, we assume that contribution of phenylalanine to protein is 4%.^44^ For example, L-[ring-^2^H_5_]-phenylalanine and L-[ring-^2^H_2_]-tyrosine are primed continuously infused. To appropriately reach isotopic equilibrium of L-[ring-^2^H_4_]-tyrosine derived from L-[ring-^2^H_5_]-phenylalanine infused (via hydroxylation reaction), a priming dose of L-[ring-^2^H_4_]-tyrosine is also administered. Blood samples are collected before the tracer infusion and after reaching *E*_*p*_. *R*_*a*_ phenylalanine and *R*_*a*_ tyrosine are determined as *F* divided by *E*_*p*_. Hydroxylation rate is determined as fraction of *R*_*a*_ tyrosine derived from phenylalanine times *R*_*a*_ tyrosine. Using these kinetic values (that is, *R*_*a*_ tracee and hydroxylation rate), we can calculate the rate of protein synthesis (*PS*), protein breakdown (*PB*), and thus net protein balance (*NB*=*PS*−*PB*) at the whole-body level. Because fates of *R*_*d*_ phenylalanine (*R*_*a*_=*R*_*d*_ at a steady state) are PS and hydroxylation to tyrosine, PS is calculated as follows: subtraction of the hydroxylation rate from *R*_*d*_ phenylalanine, then divided by 0.04, reflecting the fractional contribution of phenylalanine to protein.^44^ *PB* is directly reflected by *R*_*a*_ phenylalanine divided by 0.04. Finally, *NB* is the balance between *PS* and *PB* (that is, *NB*=*PS*–*PB*). It is important to note that *MPE* must be used for calculations of all the components of PS because both tracee and tracer are used for the process of synthesis, whereas *TTR* must be used for calculations of those of PB because tracee, but not tracer, is derived from PB. Second, protein kinetics can be quantified at the tissue (for example, muscle) level. Using the tracer incorporation method, the *FSR* and the fractional breakdown rate of tissue proteins are accessible but not at the same time frame. It is possible to determine simultaneously both protein synthesis and breakdown at the organ level with an arteriovenous balance technique and stable isotope tracers.^33, 44, 46, 47^ However, this method has inherent invasiveness and needs the determination of regional blood flow, which is often variable.^33, 46, 47, 48^ Muscle protein synthesis rate can be measured as the *FSR* using the precursor-product method.^26^ The method can also be used for the determination of individual protein *FSR* after extracting the protein of interest,^49^ but not for fractional breakdown rate because originations (specific protein) of products (AAs) for fractional breakdown rate are uncertain. Protein *FSR* is the fraction of the total protein pool size that has been newly synthesized in a given time. The *FSR* method is not limited to protein in its application but also to others including DNA, which reflects the cell division rate.^29, 50^ Because *FSR* is a fractional term, the determination of the absolute synthesis rate requires knowledge of the pool size (that is, *FSR* × pool size). Because the pool size is generally constant throughout the time period of a typical metabolic study (typically ⩽8 h) for slowly turning-over compounds such as muscle protein, *FSR* can be used for direct comparisons. Exemplary calculations of muscle protein *FSR* are shown in Supplementary Data (S5). Protein *FSR* is calculated typically as changes in a representative EAA isotopic enrichment in bound proteins between two time points divided by the mean precursor enrichment of the representative EAA.^1^ Because of the difficulties in accessing true precursor enrichment (that is, tRNA charged with AAs),^51, 52^ it is common to use surrogate precursor enrichment such as intracellular^26^ or plasma AA enrichment.^6^ Plasma AA enrichment is more appropriate when the protocol involves both fasted and fed periods.^1, 6^

### Substrate oxidation

Quantifying rates of oxidation of substrates such as glucose, fatty acids and AA has long been of interest. For example, alterations in substrate oxidation such as reduced capacity to oxidize fatty acids are linked to various metabolic disorders such as insulin resistance.^53, 54^ Stable isotope tracers in conjunction with traditional indirect calorimetry (for the determination of volume of carbon dioxide produced per unit time, *VCO*_*2*_) have been used to determine the rates of oxidation of specific components of carbohydrate or lipid as well as of protein at various metabolic conditions, including at rest or during exercise at various intensities in healthy^5, 7^ or in clinical conditions.^55, 56^ The determination of the substrate oxidation rate is based on the incorporation into CO_2_ (^12^CO_2_ from tracee and ^13^CO_2_ from tracer) from carbons of substrate of interest such as glucose,^14, 18^ free fatty acids (for example, palmitate)^18, 57^ or AA (for example, leucine).^17^ Exemplary calculations of FFA oxidation using a ^13^C-labeled palmitate tracer are shown in Supplementary Data (S6). A ^13^C-labeled tracer is primed continuously infused, and blood samples (for the determination of plasma enrichment of substrate of interest) and breath samples (for the determination of breath CO_2_ enrichment) are obtained before the tracer infusion and after plateau enrichment is achieved. From this, the following information can be obtained: (i) percent of substrate uptake oxidized via Krebs cycle (% Uptake Oxidized), (ii) substrate oxidation rate and (iii) percent of total CO_2_ production from oxidation of substrate of interest (% CO_2_ from oxidation). For example, to determine the rate of palmitate oxidation in a fasted resting state, a ^13^C-labeled palmitate tracer (for example, 1-^13^C-palmitate) can be infused via the primed continuous infusion method (Supplementary Data (S6)). Breath and plasma samples are obtained before start of the tracer infusion and then at isotopic enrichment, from which tracer enrichments are determined. Calculation of the following steady-state kinetics is possible: % uptake oxidized can be calculated as labeled CO_2_ enrichment from breath sample times total CO_2_ production (determined by indirect calorimetry) divided by plasma enrichment of the tracer infused. In a steady state, *R*_*a*_ palmitate is equal to *R*_*d*_ palmitate, which can be determined as the infusion rate of tracer (*F*) divided by plateau enrichment (*E*_*p*_). Using this information, palmitate oxidation rate can be calculated as *R*_*d*_ palmitate multiplied by % uptake oxidized divided by 100. If a multiple-labeled tracer is used, correction must be made by dividing the value by the number of labeled carbons when the results are expressed as mmol min^−1^. To calculate total FFA oxidation rate, the rate of palmitate oxidation is divided by the fraction of palmitate in total fatty acids. As not all the carbon produced at the cellular level appears in the circulation because of CO_2_ retention in the body, as well as loss of carbon label via isotopic exchange in Krebs cycle, a correction of the rate of ^13^CO_2_ should be made, which is typically performed by dividing fractional recovery of ^13^CO_2_ obtained from infused carbon labeled acetate.^58, 59^

### Summary

Application of stable isotope tracers in metabolic studies in humans enables the quantification of *in vivo* metabolic kinetics in both normal and pathophysiological circumstances. The use of stable isotope tracers are not limited to human studies, but can be used successfully in animal and *in vitro* models as well. However, given the safety of the methodology, it is particularly well suited for clinical studies in human subjects. Stable isotope tracers can be successfully used in conjunction with molecular and cellular biology tools, thereby providing an in-depth dynamic assessment of metabolic changes, as well as simultaneous investigation of the molecular basis for the observed kinetic responses.
