# Supplementary material for: Applications of stable, nonradioactive isotope tracers in in vivo human metabolic research
Source: Exp Mol Med. 2016 Jan 15;48(1):e203–. doi: 10.1038/emm.2015.97 (PMC4686699; doi:10.1038/emm.2015.97)
Supplement: Supplementary Information [file emm201597x1.doc]

**Supplemental Data:**

**Exemplary calculations of isotopic enrichment and *in vivo* kinetics**

| **S1. Calculation** **of isotope enrichment**  Instrument: GC/MS  Tracer: 6,6-^2^H_2_-glucose (100%)*^1^*  Ionization method: chemical impact ionization (CI)  Derivatization method*^2^*: pentaacetate derivatization  Determination of Enrichment  Enrichment before tracer infusion  Abundance at *M+0* (*m/z*, 331): 2213121  Abundance at *M+2* (*m/z*, 333): 67498  Background *TTR* (*M+2/M+0*) = 67498 / 2213121 = 0.0304990  Enrichment at isotopic equilibrium  Abundance at *M+0* (*m/z*, 331): 2783834  Abundance at *M+2* (*m/z*, 333): 204673  Sample *TTR* (*M+2/M+0*) = 204673 / 2783834 = 0.0735220  Background subtracted enrichment  *TTR* = Sample *TTR* – Background *TTR* = 0.0735220 – 0.0304990 = 0.043023  *MPE* = *TTR* / (1 + *TTR*) x 100 = (0.043023 / (1 + 0.043023)) x 100 = 4.1248%  *^1^* Assumed that 100% is 6,6-^2^H_2_-glucose, but it is typically lower than 100%, which needs to be taken account for correct calculations.  *^2^* Derivatization process of glucose with pentaacetate added many atoms, which thus *m/z* of those glucose ions are much heavier than 180. |
| --- |

| **S2. Calculation of rates of appearance and disappearance of glucose**  Instrument: GC/MS  Tracers: 1-^13^C-glucose (prime: 17 μmol/kg and rate: 0.22 μmol/kg/min)  Calculation of *MPE*  Background *MPE* (*M+1*/*M+0*): 6.12328%  Sample *MPE* (*M+1*/*M+0*): 8.20123%  (Sample – Background) *MPE*: 8.20123% - 6.12328% = 2.10212%  Therefore, *MPE* = 2.10212%  Calculations of tracee kinetics*^1^*  1. *R_a_* glucose = *F* / *E_p_* = 0.22 μmol/kg/min / (2.10212% / 100) = 10.47 μmol/kg/min  Because *R_a_* glucose equals to *R_d_* glucose in a steady state,  2. *R_d_* glucose = 10.47 μmol/kg/min  *^1^* Note that while *TTR* (or *t/T*) is expressed as a ratio, *MPE* or *APE* is expressed in % (i.e., *TTR* / (*TTR* + 1) x 100). This difference must be taken account for the calculation of kinetics. |
| --- |

| **S3. Calculation of lipolytic rate and fatty acid cycling**  Instrument: GC/MS  Tracers:  1-^13^C-palmiate*^1^* (rate: 0.04 μmol/kg/min)  [1,1,2,3,3-^2^H_5_]glycerol*^2^* (prime: 1.2 μmol/kg and rate: 0.08 μmol/kg/min)  Enrichment at isotopic equilibrium (*E_p_*) (background subtracted):  *TTR* (plasma palmitate): 0.035  *TTR* (plasma glycerol): 0.040  Calculations of tracee kinetics  1. *R_a_* palmitate = *F* / *E_p_* = 0.04 μmol/kg/min /0.035 = 1.143 μmol/kg/min  2. *R_a_* FFA = Ra palmitate (μmol/kg/min) / FC*^3^*  3. *R_a_* glycerol (lipolysis) = *F* / *E_p_* = 0.08 μmol/kg/min / 0.040 = 2.0 μmol/kg/min  4. Intracellular recycling (re-esterification): *R_a_* glycerol x 3 – *R_a_* FFA  = 2.0 μmol/kg/min x 3 – 1.143 μmol/kg/min/0.65 = 4.242 μmol/kg/min  5. Extracellular recycling: *R_a_* FFA – FFA oxidation rate (See Example 6)  6. Total recycling = intracellular + extracellular recycling = *R_a_* glycerol x 3 – FFA oxidation  *^1^* Palmitate tracer was continuously infused without a priming dose because of the rapidly mixing plasma pool and the rapid turnover rate of plasma FFA, so that *E_p_* is achieved in a shorter time.^1^  *^2^* Glycerol tracer, in which 2 hydrogens are attached to 1-carbon and 3-carbon positions, whereas 1 hydrogen is attached to 2-carbon position.  *^3^* FC, fractional contribution of palmitate to total FFA = 0.65 at rest.^2^ |
| --- |

| **S4. Calculation of whole body protein kinetics**  Instrument: GC/MS  Tracers:  L-[ring-^2^H_5_]phenylalnine (prime, 3.07 μmol/kg; rate, 0.084 μmol/kg/min)  L-[ring-^2^H_2_]tyrosine (prime, 3.07 μmol/kg; rate, 0.033 μmol/kg/min)  L-[ring-^2^H_4_]tyrosine (prime, 0.30 μmol/kg)  Enrichment at isotopic equilibrium (*E_p_*) (background subtracted):  Plasma phenylalanine (*M+5*/*M+0*): *TTR*, 0.0941; *MPE*, 8.6007%  Plasma phenylalanine (*M+4*/*M+0*): *TTR*, 0.0241; *MPE*, 2.3533%  Plasma tyrosine (*M+2*/*M+0*): *TTR*, 0.0442; *MPE*, 4.2329%  Calculations of tracee kinetics  1. *R_a_* phenylalanine (*R_a_* Phe)  *R_a_* Phe_TTR_ = *F* / *E_TTR_* = 0.084 μmol/kg/min / 0.0941 = 0.8927 μmol/kg/min  *R_a_* Phe_MPE_ = *F* / *E_MPE_* = 0.084 μmol/kg/min / (8.6007 / 100)= 0.9767 μmol/kg/min  2. *R_a_* phenylalanine (*R_a_* Tyr)  *R_a_* Tyr_TTR_ = *F* / *E_TTR_* = 0.033 μmol/kg/min / 0.0442 = 0.747 μmol/kg/min  *R_a_* Tyr_MPE_ = *F* / *E_MPE_* = 0.033 μmol/kg/min / (4.2329 / 100) = 0.7796 μmol/kg/min  3. Fractional *R_a_* of Tyr from Phe (*MPE*) = *E_TYR M+4_* / *E_PHE M+5_* = 2.3533% / 8.6007% = 0.2736  4. Phe hydroxylation rate = Fractional *R_a_* of Tyr from Phe x *R_a_* Tyr_MPE_ = 0.2736 X 0.7796 μmol/kg/min = 0.2133 μmol/kg/min  5. Rate of protein synthesis (*PS*) = (*R_a_* phe_MPE_ – Phe hydroxylation rate) / Fractional contribution of Phe to protein*^1^* = (0.9767 - 0.2133) μmol/kg/min / 0.04 = 19.085 μmol/kg/min  6. Rate of protein breakdown (*PB*) = *R_a_* Phe_TTR_ / 0.04 = 0.8927 μmol/kg/min / 0.04*^a^* = 22.32 μmol/kg/min  7. Rate of net protein balance = *PS* – *PB* = (19.085 - 22.318) μmol/kg/min = -3.233 μmol/kg/min  *^1^* Assumed that fractional contribution of phenylalanine to protein is 0.04.^3^ |
| --- |

| **S5. Calculation of muscle protein fractional synthesis rate**  Instrument: GC/MS  Tracer: L-[ring-^2^H_5_]phenylalnine (prime, 3.07 μmol/kg; rate, 0.084 μmol/kg/min)  Enrichment (background subtracted)  *MPE* (Bound protein: product (phenylalanine) enrichment, *E_B_*):  *t*_1_ (120 min): 0.0323% (*M+5*/*M+0*)  *t*_2_ (420 min): 0.0586% (*M+5*/*M+0*)  *Delta MPE:* (0.0586 - 0.0323)% = 0.0262%  *MPE* (Intracellular amino acids: precursor (phenylalanine) enrichment, *E_IC_*):  *t*_1_ (120 min): 7.6277% (*M+5*/*M+0*)  *t*_2_ (360 min): 7.7047% (*M+5*/*M+0*)  *Average MPE: (*7.6277 + 7.7047) / 2 = 7.6662%  Calculations of tracee kinetics  *FSR* (%•h^-1^) = [*E_B_* (t_2_) – *E_B_* (t_1_)] / [(*E_IC_* (t_2_) + *E_IC_* (t_1_)) / 2 x time (min)] / 60 min/hour x 100  = (0.0262 / 100) / (7.6662 / 100 X 240 min) / 60min/hour x 100 = 0.08544%/h |
| --- |

| **S 6. Calculation of substrate oxidation**  Instrument: GC/MS and IRMS  Tracer: 1-^13^C-palmiate (rate: 0.04 μmol/kg/min)  Rate of CO_2_ production (*VCO_2_*) determined using indirect calorimetry: 100 μmol/kg/min  Enrichment at isotopic equilibrium (*E_p_*) (background subtracted):  Breath *APE* (by IRMS)  *APE* (^13^CO_2_/^12^CO_2_): 0.00012  Acetate correction factor: 0.6  Plasma (by GC/MS)  *MPE* (plasma palmitate, *M+1*/*M+0*): 0.033  Calculations of tracee kinetics  1. % Uptake Oxidized: [*MPE* (CO_2_) X *VCO_2_* / (Infusion rate of ^13^C x Acetate correction factor)] x 100 = (0.00012 x 100 μmol/kg/min) / (0.04 μmol/kg/min x 0.6) X 100 = 50%  2. *R_d_* palmitate = *F* / *E_p_* = 0.04 μmol/kg/min / 0.033 = 1.212 μmol/kg/min  3. Oxidation rate = *R_d_* palmitate x % uptake oxidized / 100 = *F* / *E_p_* x % Uptake Oxidized / 100 = 1.212 μmol/kg/min x 0.5 = 0.606 μmol/kg/min  4. % Oxidation from tracee = (Oxidation rate x μmol CO_2_/μmol palmitate) / *VCO_2_* x 100 = [(0.606 μmol/kg/min x 16 μmol CO_2_/μmol palmitate) / 100 μmol/kg/min] x 100 = 9.696% |
| --- |

**References:**

1 Greenough WB, Crespin SR, Steinberg D. Infusion of long-chain fatty acid anions by continuous-flow centrifugation. *J Clin Invest* 1969; **48**: 1923–1933.

2 Mazzeo RS, Brooks GA, Schoeller DA, Budinger TF. Disposal of blood [1-13C]lactate in humans during rest and exercise. *J Appl Physiol* 1986; **60**: 232–241.

3 Biolo G, Fleming RY, Maggi SP, Wolfe RR. Transmembrane transport and intracellular kinetics of amino acids in human skeletal muscle. *Am J Physiol* 1995; **268**: E75–84.
